# Supplementary material for: Older adults demonstrate interlimb transfer of reactive gait adaptations to repeated unpredictable gait perturbations
Source: GeroScience. 2019 Nov 27;42(1):39–49. doi: 10.1007/s11357-019-00130-x (PMC7031170; doi:10.1007/s11357-019-00130-x)
Supplement: Supplementary file 1 — (PDF 1049 kb) [file 11357_2019_130_MOESM1_ESM.pdf]

Supplementary Material to

Older adults demonstrate interlimb transfer of reactive gait adaptations to repeated unpredictable gait perturbations

GeroScience

Christopher McCrum[1,2]*, Kiros Karamanidis[3], Lotte Grevendonk[1], Wiebren Zijlstra[2], Kenneth Meijer[1]

[1]Department of Nutrition and Movement Sciences, NUTRIM School of Nutrition and Translational Research in Metabolism, Maastricht University Medical Centre+, Maastricht, The Netherlands
[2]Institute of Movement and Sport Gerontology, German Sport University Cologne, Germany
[3]Sport and Exercise Science Research Centre, School of Applied Sciences, London South Bank University, London, UK

*Correspondence:
Christopher McCrum
Department of Nutrition and Movement Sciences
Maastricht University,
PO Box 616, Maastricht, 6200 MD, The Netherlands
chris.mccrum@maastrichtuniversity.nl
+31 (0)43 388 1621

eMethods

For this study, we used the margins of stability (MoS) concept (Hof et al. 2005), one of only a few clearly defined and accepted measures of the mechanical stability of the body configuration during gait (Bruijn et al. 2013), useful for assessing changes in gait stability due to mechanical perturbations and balance loss. The anteroposterior MoS (MoS_{AP}) were calculated at foot touchdown as the difference between the anterior boundary of the base of support (anteroposterior component of the hallux marker projection to the ground) and the extrapolated centre of mass as defined by Hof et al. (2005), adapted for our reduced kinematic model based on Süptitz et al. (2013), as follows:

$$X_{CoM} = \frac{P_{TroL} + P_{TroR}}{2} - P_{HalluxP} + \frac{0.5\left(\frac{V_{TroL} + V_{TroR}}{2} + V_{C7}\right) + |V_{Belt}|}{\sqrt{\frac{g}{L_{Ref}}}}$$

where P_{TroL}, P_{TroR} and $P_{HalluxP}$ are the trochanter and the posterior hallux marker anteroposterior positions respectively; V_{TroL} , V_{TroR} and V_{C7} are the anteroposterior velocities of the trochanter and C7 markers respectively; V_{Belt} is the treadmill belt velocity; g is gravitational acceleration (9.81m/s^2); and L_{Ref} is the reference leg length. This reduced kinematic model was previously shown to be suitable for assessing the MoS and it's components during unperturbed and perturbed treadmill walking in young, middle and older-aged healthy adults, with high correlations and no clear differences compared to a full kinematic model (Süptitz et al. 2013).

eResults

Regarding the MoS_{ML}, the two way repeated measures ANOVAs for $Pert1_R$, $Pert2_L$ and $Pert9_L$ revealed a significant age effect for $Pert1_R$ and $Pert9_L$, but not for $Pert2_L$ ($Pert1_R$: $F_{(1, 55)}$=4.973, P=0.0298, η_p^2=0.083; $Pert2_L$: $F_{(1, 56)}$=2.031, P=0.1597, η_p^2=0.035; $Pert9_L$: $F_{(1, 56)}$=4.110, P=0.0474, η_p^2=0.068; eFig. 1). Significant Age Group by Step interactions were found for $Pert1_R$, $Pert2_L$ and $Pert9_L$ ($Pert1_R$: $F_{(9, 495)}$=1.965, P=0.0415, η_p^2=0.034; $Pert2_L$: $F_{(9, 504)}$=2.254, P=0.0177, η_p^2=0.039; $Pert9_L$: $F_{(9, 504)}$=3.610, P=0.0002, η_p^2=0.061; eFig. 1). Bonferroni tests for multiple comparisons are indicated in eFig. 1 and revealed two significant difference between the age groups at Post4 during $Pert1_R$, and significant differences at Post5 and Post6 during $Pert9_L$ but no major differences were identified pre and post repetition of the left limb perturbations, with both age groups showing significantly increased MoS_{ML} at Post2 and Post3 during $Pert2_L$ and $Pert9_L$. Complete Bonferroni results can be found in eTables 7 and 8.

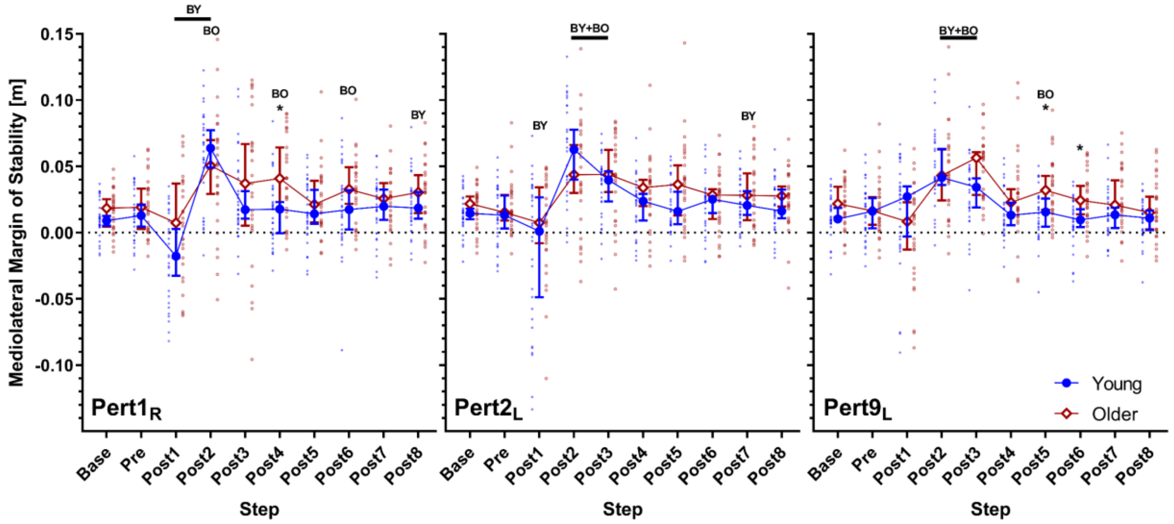

eFigure 1: Median and 95% confidence intervals (with individual data points) of the mediolateral margin of stability during the first, second and ninth perturbations ($Pert1_R$, $Pert2_L$, and $Pert9_L$, respectively) including unperturbed walking prior to each perturbation (Base), the final step prior to each perturbation (Pre) and the first eight recovery steps following the perturbations (Post1-8) for young and older adults. BO and BY: Significant difference to Base for older and young adults, respectively (P<0.05). *: Significant difference between young and older adults (P<0.05).

Regarding the investigation of the potential influence of the single trial effect on the interlimb transfer results in the older adults, a two-way repeated measures ANOVA with perturbation number ($Pert2_L$ and $Pert3_L$) and step (Base, Pre, Post1-Post8) as factors with post hoc Bonferroni tests for multiple comparisons were conducted for MoS_{AP}. Similar to the interlimb analysis, no significant perturbation number effect was found ($F_{(1, 26)}$=1.681, P=0.2061), although the size of the effect (η_p^2=0.061) was smaller than that of the interlimb analysis (η_p^2=0.092). Bonferroni tests for multiple comparisons revealed that during $Pert2_L$, the older adults did not return to MoS_{AP} values not significantly different to Base during the eight analysed recovery steps, whereas for $Pert3_L$ this was achieved at Post7, resulting in an average of two steps improvement (eFig. 2). Complete Bonferroni results can be found in eTables 17 and 18.

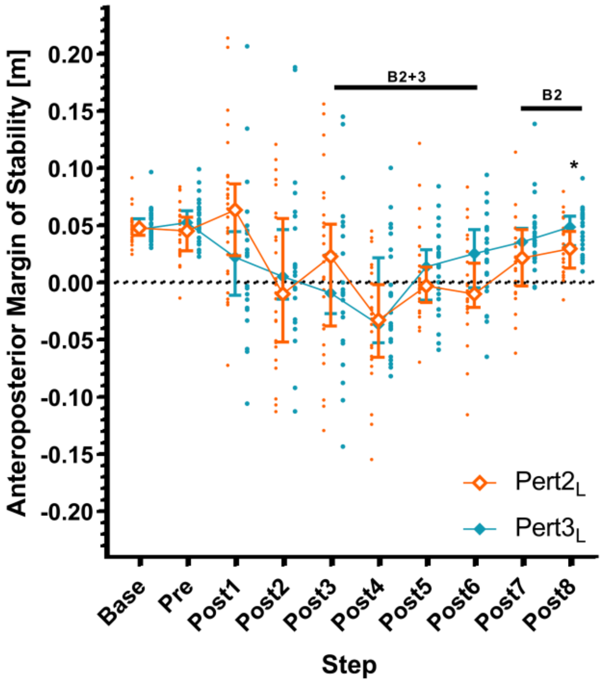

eFigure 2: Median and 95% confidence intervals (with individual data points) of the anteroposterior margin of stability during the second and third perturbations ($Pert2_L$ and $Pert3_L$, respectively) including unperturbed walking prior to each perturbation (Base), the final step prior to each perturbation (Pre) and the first eight recovery steps following the perturbations (Post1-8) for the older adults. B2 and B3: Significant difference to Base for $Pert2_L$ and $Pert3_L$, respectively (P<0.05). *: Significant difference between $Pert2_L$ and $Pert3_L$ (P<0.05).

eTable 1: Bonferroni's multiple comparison test results comparing recovery steps to baseline in $Pert1_R$, $Pert2_L$ and $Pert9_L$ for MoS_{AP} [m] in the young and older adults

Perturbation	Test details	Mean 1	Mean 2	Mean Diff.	SE of diff.	N1	N2	t	DF	95.00% CI of diff.	Adjusted P Value
**$Pert1_R$**	Young										
	Base vs. Pre	0.04649	0.04944	-0.002954	0.003325	30	30	0.8884	29	-0.01292 to 0.007008	>0.9999
	Base vs. Post1	0.04649	-0.01941	0.0659	0.009999	30	30	6.59	29	0.03594 to 0.09585	<0.0001
	Base vs. Post2	0.04649	-0.04436	0.09085	0.01635	30	30	5.556	29	0.04186 to 0.1398	<0.0001
	Base vs. Post3	0.04649	-0.06068	0.1072	0.01102	30	30	9.721	29	0.07414 to 0.1402	<0.0001
	Base vs. Post4	0.04649	-0.03958	0.08607	0.009342	30	30	9.213	29	0.05808 to 0.1141	<0.0001
	Base vs. Post5	0.04649	0.00193	0.04456	0.0101	30	30	4.411	29	0.01429 to 0.07482	0.0012
	Base vs. Post6	0.04649	0.02455	0.02194	0.008469	30	30	2.591	29	-0.003432 to 0.04732	0.1335
	Base vs. Post7	0.04649	0.03039	0.0161	0.006845	30	30	2.351	29	-0.004411 to 0.03660	0.2313
	Base vs. Post8	0.04649	0.03976	0.006733	0.005854	30	30	1.15	29	-0.01080 to 0.02427	>0.9999
	Older										
	Base vs. Pre	0.0496	0.04576	0.003836	0.003399	27	27	1.128	26	-0.006443 to 0.01411	>0.9999
	Base vs. Post1	0.0496	0.1117	-0.06213	0.01244	27	27	4.996	26	-0.09973 to -0.02452	0.0003
	Base vs. Post2	0.0496	0.02267	0.02693	0.01389	27	27	1.939	26	-0.01506 to 0.06892	0.5709
	Base vs. Post3	0.0496	-0.0007875	0.05038	0.01063	27	27	4.739	26	0.01824 to 0.08253	0.0006
	Base vs. Post4	0.0496	-0.02787	0.07746	0.01084	27	27	7.143	26	0.04467 to 0.1103	<0.0001
	Base vs. Post5	0.0496	-0.009746	0.05934	0.01015	27	27	5.846	26	0.02865 to 0.09004	<0.0001
	Base vs. Post6	0.0496	0.004204	0.04539	0.007151	27	27	6.348	26	0.02377 to 0.06702	<0.0001
	Base vs. Post7	0.0496	0.01565	0.03395	0.007473	27	27	4.542	26	0.01135 to 0.05654	0.001
	Base vs. Post8	0.0496	0.02613	0.02347	0.0077	27	27	3.048	26	0.0001856 to 0.04675	0.0471
**$Pert2_L$**	Young										
	Base vs. Pre	0.04342	0.03979	0.003637	0.002281	30	30	1.594	29	-0.003198 to 0.01047	>0.9999
	Base vs. Post1	0.04342	-0.01938	0.06281	0.01185	30	30	5.299	29	0.02730 to 0.09831	<0.0001
	Base vs. Post2	0.04342	-0.02011	0.06353	0.01373	30	30	4.626	29	0.02239 to 0.1047	0.0006
	Base vs. Post3	0.04342	-0.03096	0.07438	0.01267	30	30	5.87	29	0.03642 to 0.1123	<0.0001
	Base vs. Post4	0.04342	-0.03964	0.08307	0.009214	30	30	9.016	29	0.05546 to 0.1107	<0.0001
	Base vs. Post5	0.04342	0.00629	0.03713	0.009356	30	30	3.969	29	0.009103 to 0.06516	0.0039
	Base vs. Post6	0.04342	0.0266	0.01682	0.005298	30	30	3.175	29	0.0009481 to 0.03269	0.0318
	Base vs. Post7	0.04342	0.04451	-0.001082	0.004687	30	30	0.2309	29	-0.01512 to 0.01296	>0.9999
	Base vs. Post8	0.04342	0.0363	0.007127	0.003821	30	30	1.865	29	-0.004321 to 0.01857	0.6509
	Older										
	Base vs. Pre	0.0478	0.04399	0.003813	0.004662	28	28	0.8179	27	-0.01024 to 0.01786	>0.9999
	Base vs. Post1	0.0478	0.06765	-0.01985	0.01443	28	28	1.375	27	-0.06334 to 0.02365	>0.9999
	Base vs. Post2	0.0478	0.002104	0.0457	0.01349	28	28	3.388	27	0.005044 to 0.08635	0.0196
	Base vs. Post3	0.0478	0.01392	0.03388	0.01371	28	28	2.471	27	-0.007437 to 0.07520	0.1806
	Base vs. Post4	0.0478	-0.03748	0.08529	0.01072	28	28	7.954	27	0.05297 to 0.1176	<0.0001

	Base vs. Post5	0.0478	0.001129	0.04667	0.008095	28	28	5.765	27	0.02228 to 0.07107	<0.0001
	Base vs. Post6	0.0478	-0.005296	0.0531	0.007842	28	28	6.771	27	0.02946 to 0.07673	<0.0001
	Base vs. Post7	0.0478	0.01784	0.02997	0.006037	28	28	4.964	27	0.01177 to 0.04816	0.0003
	Base vs. Post8	0.0478	0.0303	0.0175	0.004551	28	28	3.845	27	0.003782 to 0.03121	0.006
**Pert9$_L$**	Young										
	Base vs. Pre	0.05127	0.04608	0.005184	0.003708	30	30	1.398	29	-0.005926 to 0.01629	>0.9999
	Base vs. Post1	0.05127	-0.04829	0.09956	0.008349	30	30	11.92	29	0.07454 to 0.1246	<0.0001
	Base vs. Post2	0.05127	-0.01154	0.06281	0.01071	30	30	5.864	29	0.03072 to 0.09490	<0.0001
	Base vs. Post3	0.05127	0.01292	0.03835	0.01047	30	30	3.663	29	0.006980 to 0.06972	0.0089
	Base vs. Post4	0.05127	0.01281	0.03846	0.007465	30	30	5.151	29	0.01609 to 0.06082	0.0001
	Base vs. Post5	0.05127	0.03754	0.01373	0.005653	30	30	2.429	29	-0.003205 to 0.03067	0.1941
	Base vs. Post6	0.05127	0.04199	0.009278	0.00488	30	30	1.901	29	-0.005343 to 0.02390	0.6054
	Base vs. Post7	0.05127	0.05671	-0.005442	0.005333	30	30	1.02	29	-0.02142 to 0.01053	>0.9999
	Base vs. Post8	0.05127	0.05212	-0.0008489	0.003863	30	30	0.2198	29	-0.01242 to 0.01072	>0.9999
	Older										
	Base vs. Pre	0.05456	0.05559	-0.001034	0.0039	28	28	0.2651	27	-0.01279 to 0.01072	>0.9999
	Base vs. Post1	0.05456	-0.02543	0.07999	0.01349	28	28	5.93	27	0.03934 to 0.1206	<0.0001
	Base vs. Post2	0.05456	0.01246	0.0421	0.01043	28	28	4.038	27	0.01068 to 0.07352	0.0036
	Base vs. Post3	0.05456	0.004749	0.04981	0.01065	28	28	4.679	27	0.01773 to 0.08189	0.0006
	Base vs. Post4	0.05456	0.01211	0.04245	0.009851	28	28	4.309	27	0.01276 to 0.07214	0.0018
	Base vs. Post5	0.05456	0.02975	0.02481	0.008151	28	28	3.044	27	0.0002450 to 0.04937	0.0464
	Base vs. Post6	0.05456	0.03516	0.0194	0.006734	28	28	2.881	27	-0.0008939 to 0.03969	0.0691
	Base vs. Post7	0.05456	0.04977	0.004786	0.003699	28	28	1.294	27	-0.006363 to 0.01594	>0.9999
	Base vs. Post8	0.05456	0.04842	0.006134	0.00423	28	28	1.45	27	-0.006613 to 0.01888	>0.9999

eTable 2: Bonferroni's multiple comparison test results comparing the young and older adults in $Pert1_R$, $Pert2_L$ and $Pert9_L$ for MoS_{AP} [m]

Perturbation	Test details	Mean 1	Mean 2	Mean Diff.	SE of diff.	N1	N2	t	DF	95.00% CI of diff.	Adjusted P Value
**$Pert1_R$**	Young - Older										
	Base	0.04649	0.0496	-0.003109	0.00317	30	27	0.9805	53.23	-0.01239 to 0.006177	>0.9999
	Pre	0.04944	0.04576	0.003681	0.005625	30	27	0.6545	54.19	-0.01278 to 0.02014	>0.9999
	Post1	-0.01941	0.1117	-0.1311	0.01545	30	27	8.488	51.21	-0.1765 to -0.08581	<0.0001
	Post2	-0.04436	0.02267	-0.06703	0.02173	30	27	3.085	54.55	-0.1306 to -0.003460	0.0319
	Post3	-0.06068	-0.0007875	-0.0599	0.01492	30	27	4.014	54.99	-0.1035 to -0.01626	0.0018
	Post4	-0.03958	-0.02787	-0.01171	0.01481	30	27	0.7908	52.49	-0.05511 to 0.03169	>0.9999
	Post5	0.00193	-0.009746	0.01168	0.01426	30	27	0.8191	54.99	-0.03002 to 0.05337	>0.9999
	Post6	0.02455	0.004204	0.02034	0.01151	30	27	1.768	54.9	-0.01331 to 0.05400	0.8262
	Post7	0.03039	0.01565	0.01474	0.009696	30	27	1.52	54.19	-0.01363 to 0.04312	>0.9999
	Post8	0.03976	0.02613	0.01363	0.009728	30	27	1.401	48.45	-0.01499 to 0.04224	>0.9999
**$Pert2_L$**	Young - Older										
	Base	0.04342	0.0478	-0.004378	0.003218	30	28	1.36	50.76	-0.01382 to 0.005067	>0.9999
	Pre	0.03979	0.04399	-0.004202	0.005268	30	28	0.7978	49.01	-0.01969 to 0.01128	>0.9999
	Post1	-0.01938	0.06765	-0.08703	0.01795	30	28	4.848	53.7	-0.1396 to -0.03447	0.0001
	Post2	-0.02011	0.002104	-0.02221	0.01918	30	28	1.158	55.98	-0.07827 to 0.03384	>0.9999
	Post3	-0.03096	0.01392	-0.04488	0.01904	30	28	2.358	55.04	-0.1006 to 0.01080	0.2198
	Post4	-0.03964	-0.03748	-0.002159	0.01369	30	28	0.1577	55.61	-0.04219 to 0.03787	>0.9999
	Post5	0.00629	0.001129	0.005161	0.01246	30	28	0.4143	54.94	-0.03127 to 0.04160	>0.9999
	Post6	0.0266	-0.005296	0.0319	0.009763	30	28	3.267	48.6	0.003185 to 0.06061	0.02
	Post7	0.04451	0.01784	0.02667	0.00862	30	28	3.094	49.59	0.001343 to 0.05200	0.0324
	Post8	0.0363	0.0303	0.005992	0.005649	30	28	1.061	54.39	-0.01054 to 0.02252	>0.9999
**$Pert9_L$**	Young - Older										
	Base	0.05127	0.05456	-0.00329	0.003844	30	28	0.856	50.37	-0.01457 to 0.007995	>0.9999
	Pre	0.04608	0.05559	-0.009507	0.005474	30	28	1.737	54.12	-0.02553 to 0.006512	0.8808
	Post1	-0.04829	-0.02543	-0.02285	0.01568	30	28	1.458	43.13	-0.06924 to 0.02353	>0.9999
	Post2	-0.01154	0.01246	-0.024	0.01445	30	28	1.661	55.82	-0.06623 to 0.01823	>0.9999
	Post3	0.01292	0.004749	0.008169	0.01474	30	28	0.554	54.68	-0.03496 to 0.05130	>0.9999
	Post4	0.01281	0.01211	0.0007005	0.01152	30	28	0.06081	51.34	-0.03309 to 0.03449	>0.9999
	Post5	0.03754	0.02975	0.007787	0.009943	30	28	0.7831	45.95	-0.02153 to 0.03711	>0.9999
	Post6	0.04199	0.03516	0.006832	0.008266	30	28	0.8266	51.67	-0.01741 to 0.03107	>0.9999
	Post7	0.05671	0.04977	0.006938	0.007204	30	28	0.963	55.17	-0.01413 to 0.02800	>0.9999
	Post8	0.05212	0.04842	0.003693	0.006066	30	28	0.6088	54.88	-0.01405 to 0.02143	>0.9999

eTable 3: Bonferroni's multiple comparison test results comparing recovery steps to baseline in Pert1$_R$, Pert2$_L$ and Pert9$_L$ for BoS [m] in the young and older adults

Perturbation	Test details	Mean 1	Mean 2	Mean Diff.	SE of diff.	N1	N2	t	DF	95.00% CI of diff.	Adjusted P Value
**Pert1$_R$**	Young										
	Base vs. Pre	0.6682	0.6668	0.001409	0.003597	30	30	0.3916	29	-0.009367 to 0.01218	>0.9999
	Base vs. Post1	0.6682	0.7997	-0.1315	0.01834	30	30	7.173	29	-0.1865 to -0.07660	<0.0001
	Base vs. Post2	0.6682	0.3485	0.3197	0.03274	30	30	9.765	29	0.2216 to 0.4178	<0.0001
	Base vs. Post3	0.6682	0.6344	0.03379	0.02678	30	30	1.261	29	-0.04646 to 0.1140	>0.9999
	Base vs. Post4	0.6682	0.7061	-0.03789	0.01115	30	30	3.398	29	-0.07129 to -0.004487	0.0179
	Base vs. Post5	0.6682	0.6424	0.02572	0.009957	30	30	2.583	29	-0.004110 to 0.05555	0.1359
	Base vs. Post6	0.6682	0.6505	0.01764	0.008043	30	30	2.194	29	-0.006451 to 0.04174	0.3277
	Base vs. Post7	0.6682	0.6365	0.03167	0.006189	30	30	5.117	29	0.01313 to 0.05021	0.0002
	Base vs. Post8	0.6682	0.6511	0.01701	0.00671	30	30	2.536	29	-0.003088 to 0.03712	0.1518
	Older										
	Base vs. Pre	0.6438	0.64	0.003826	0.004034	27	27	0.9484	26	-0.008373 to 0.01603	>0.9999
	Base vs. Post1	0.6438	0.7432	-0.09937	0.01022	27	27	9.723	26	-0.1303 to -0.06847	<0.0001
	Base vs. Post2	0.6438	0.2436	0.4002	0.03247	27	27	12.33	26	0.3020 to 0.4984	<0.0001
	Base vs. Post3	0.6438	0.5265	0.1173	0.02718	27	27	4.318	26	0.03516 to 0.1995	0.0018
	Base vs. Post4	0.6438	0.6003	0.04344	0.02796	27	27	1.554	26	-0.04109 to 0.1280	>0.9999
	Base vs. Post5	0.6438	0.6205	0.0233	0.01868	27	27	1.248	26	-0.03317 to 0.07977	>0.9999
	Base vs. Post6	0.6438	0.6482	-0.004443	0.01213	27	27	0.3664	26	-0.04111 to 0.03222	>0.9999
	Base vs. Post7	0.6438	0.6157	0.02805	0.01354	27	27	2.071	26	-0.01290 to 0.06899	0.4355
	Base vs. Post8	0.6438	0.629	0.01479	0.01053	27	27	1.404	26	-0.01705 to 0.04663	>0.9999
**Pert2$_L$**	Young										
	Base vs. Pre	0.6635	0.6556	0.007867	0.003805	30	30	2.068	29	-0.003531 to 0.01927	0.4292
	Base vs. Post1	0.6635	0.8094	-0.1459	0.01811	30	30	8.055	29	-0.2002 to -0.09164	<0.0001
	Base vs. Post2	0.6635	0.4273	0.2362	0.03137	30	30	7.53	29	0.1422 to 0.3302	<0.0001
	Base vs. Post3	0.6635	0.637	0.02653	0.01729	30	30	1.534	29	-0.02527 to 0.07833	>0.9999
	Base vs. Post4	0.6635	0.7137	-0.0502	0.01132	30	30	4.436	29	-0.08409 to -0.01630	0.0011
	Base vs. Post5	0.6635	0.6667	-0.003187	0.008528	30	30	0.3737	29	-0.02874 to 0.02236	>0.9999
	Base vs. Post6	0.6635	0.6543	0.009177	0.007922	30	30	1.158	29	-0.01456 to 0.03291	>0.9999
	Base vs. Post7	0.6635	0.6452	0.01832	0.008316	30	30	2.203	29	-0.006596 to 0.04323	0.3215
	Base vs. Post8	0.6635	0.6536	0.009872	0.005144	30	30	1.919	29	-0.005541 to 0.02528	0.584
	Older										
	Base vs. Pre	0.6371	0.6317	0.005399	0.005054	28	28	1.068	27	-0.009831 to 0.02063	>0.9999
	Base vs. Post1	0.6371	0.7397	-0.1026	0.02012	28	28	5.1	27	-0.1632 to -0.04197	0.0002
	Base vs. Post2	0.6371	0.2684	0.3686	0.02456	28	28	15.01	27	0.2946 to 0.4426	<0.0001
	Base vs. Post3	0.6371	0.5438	0.09323	0.01554	28	28	6	27	0.04640 to 0.1401	<0.0001
	Base vs. Post4	0.6371	0.5977	0.03941	0.02513	28	28	1.568	27	-0.03633 to 0.1152	>0.9999

	Base vs. Post5	0.6371	0.6382	-0.00111	0.01373	28	28	0.0809	27	-0.04248 to 0.04026	>0.9999
	Base vs. Post6	0.6371	0.6621	-0.025	0.01198	28	28	2.087	27	-0.06110 to 0.01110	0.4183
	Base vs. Post7	0.6371	0.6355	0.001548	0.008807	28	28	0.1758	27	-0.02500 to 0.02809	>0.9999
	Base vs. Post8	0.6371	0.6335	0.00356	0.006141	28	28	0.5797	27	-0.01495 to 0.02207	>0.9999
**Pert9$_L$**	Young										
	Base vs. Pre	0.6725	0.6671	0.005362	0.00302	30	30	1.776	29	-0.003684 to 0.01441	0.7762
	Base vs. Post1	0.6725	0.7637	-0.09119	0.02338	30	30	3.899	29	-0.1612 to -0.02113	0.0047
	Base vs. Post2	0.6725	0.5826	0.08989	0.01879	30	30	4.785	29	0.03361 to 0.1462	0.0004
	Base vs. Post3	0.6725	0.6425	0.02999	0.01391	30	30	2.156	29	-0.01167 to 0.07165	0.3553
	Base vs. Post4	0.6725	0.6992	-0.02667	0.007545	30	30	3.535	29	-0.04927 to -0.004066	0.0125
	Base vs. Post5	0.6725	0.673	-0.0004707	0.00594	30	30	0.0792	29	-0.01827 to 0.01733	>0.9999
	Base vs. Post6	0.6725	0.6694	0.003134	0.004412	30	30	0.7103	29	-0.01009 to 0.01635	>0.9999
	Base vs. Post7	0.6725	0.6636	0.008934	0.004477	30	30	1.996	29	-0.004478 to 0.02235	0.4989
	Base vs. Post8	0.6725	0.6638	0.008676	0.004403	30	30	1.97	29	-0.004516 to 0.02187	0.5258
	Older										
	Base vs. Pre	0.642	0.6464	-0.004371	0.004482	28	28	0.9753	27	-0.01788 to 0.009136	>0.9999
	Base vs. Post1	0.642	0.7138	-0.07178	0.02028	28	28	3.539	27	-0.1329 to -0.01065	0.0133
	Base vs. Post2	0.642	0.5109	0.1311	0.026	28	28	5.043	27	0.05276 to 0.2095	0.0002
	Base vs. Post3	0.642	0.56	0.08201	0.01619	28	28	5.067	27	0.03323 to 0.1308	0.0002
	Base vs. Post4	0.642	0.6414	0.000607	0.01175	28	28	0.0516	27	-0.03481 to 0.03603	>0.9999
	Base vs. Post5	0.642	0.6302	0.01175	0.01323	28	28	0.8883	27	-0.02811 to 0.05161	>0.9999
	Base vs. Post6	0.642	0.6591	-0.01716	0.008674	28	28	1.978	27	-0.04330 to 0.008984	0.524
	Base vs. Post7	0.642	0.6342	0.007775	0.007322	28	28	1.062	27	-0.01429 to 0.02984	>0.9999
	Base vs. Post8	0.642	0.6454	-0.003409	0.006732	28	28	0.5064	27	-0.02370 to 0.01688	>0.9999

eTable 4: Bonferroni's multiple comparison test results comparing the young and older adults in Pert1$_R$, Pert2$_L$ and Pert9$_L$ for BoS [m]

Perturbation	Test details	Mean 1	Mean 2	Mean Diff.	SE of diff.	N1	N2	t	DF	95.00% CI of diff.	Adjusted P Value
**Pert1$_R$**	Young - Older										
	Base	0.6682	0.6438	0.02437	0.01305	30	27	1.868	43.27	-0.01423 to 0.06297	0.686
	Pre	0.6668	0.64	0.02679	0.01355	30	27	1.977	46.18	-0.01317 to 0.06674	0.5407
	Post1	0.7997	0.7432	0.05654	0.02469	30	27	2.29	53.97	-0.01574 to 0.1288	0.2598
	Post2	0.3485	0.2436	0.1049	0.04659	30	27	2.252	54.98	-0.03136 to 0.2411	0.2837
	Post3	0.6344	0.5265	0.1079	0.04081	30	27	2.644	52.64	-0.01167 to 0.2275	0.1076
	Post4	0.7061	0.6003	0.1057	0.03602	30	27	2.935	35.84	-0.002043 to 0.2135	0.0579
	Post5	0.6424	0.6205	0.02195	0.02719	30	27	0.8073	37.35	-0.05917 to 0.1031	>0.9999
	Post6	0.6505	0.6482	0.002281	0.02124	30	27	0.1074	46.4	-0.06031 to 0.06487	>0.9999
	Post7	0.6365	0.6157	0.02074	0.02275	30	27	0.912	37.41	-0.04711 to 0.08860	>0.9999
	Post8	0.6511	0.629	0.02214	0.01883	30	27	1.176	45.9	-0.03340 to 0.07769	>0.9999
**Pert2$_L$**	Young - Older										
	Base	0.6635	0.6371	0.02645	0.01339	30	28	1.975	44.29	-0.01312 to 0.06602	0.5452
	Pre	0.6556	0.6317	0.02398	0.01402	30	28	1.711	46.85	-0.01731 to 0.06528	0.9367
	Post1	0.8094	0.7397	0.06976	0.02983	30	28	2.339	54.26	-0.01753 to 0.1571	0.2307
	Post2	0.4273	0.2684	0.1588	0.04123	30	28	3.852	54.32	0.03818 to 0.2795	0.0031
	Post3	0.637	0.5438	0.09315	0.02674	30	28	3.484	50.05	0.01464 to 0.1717	0.0104
	Post4	0.7137	0.5977	0.1161	0.03218	30	28	3.607	37.28	0.02004 to 0.2121	0.009
	Post5	0.6667	0.6382	0.02853	0.02237	30	28	1.275	46.58	-0.03739 to 0.09445	>0.9999
	Post6	0.6543	0.6621	-0.007722	0.01921	30	28	0.4021	47.34	-0.06428 to 0.04883	>0.9999
	Post7	0.6452	0.6355	0.009682	0.0169	30	28	0.573	52.49	-0.03983 to 0.05920	>0.9999
	Post8	0.6536	0.6335	0.02014	0.01526	30	28	1.319	42.98	-0.02503 to 0.06531	>0.9999
**Pert9$_L$**	Young - Older										
	Base	0.6725	0.642	0.03052	0.01208	30	28	2.526	49.43	-0.004983 to 0.06601	0.1479
	Pre	0.6671	0.6464	0.02078	0.01344	30	28	1.546	47.59	-0.01879 to 0.06036	>0.9999
	Post1	0.7637	0.7138	0.04992	0.032	30	28	1.56	55.5	-0.04364 to 0.1435	>0.9999
	Post2	0.5826	0.5109	0.07175	0.03598	30	28	1.994	49.27	-0.03401 to 0.1775	0.5171
	Post3	0.6425	0.56	0.08253	0.02414	30	28	3.419	52.12	0.01177 to 0.1533	0.0123
	Post4	0.6992	0.6414	0.05779	0.01735	30	28	3.331	54.35	0.007018 to 0.1086	0.0156
	Post5	0.673	0.6302	0.04274	0.01751	30	28	2.44	45.44	-0.008942 to 0.09441	0.1865
	Post6	0.6694	0.6591	0.01022	0.01615	30	28	0.633	47.71	-0.03732 to 0.05777	>0.9999
	Post7	0.6636	0.6342	0.02936	0.01478	30	28	1.986	45.79	-0.01424 to 0.07296	0.5306
	Post8	0.6638	0.6454	0.01843	0.01492	30	28	1.236	44.75	-0.02562 to 0.06248	>0.9999

eTable 5: Bonferroni's multiple comparison test results comparing recovery steps to baseline in Pert1$_R$, Pert2$_L$ and Pert9$_L$ for X$_{CoM}$ [m] in the young and older adults

Perturbation	Test details	Mean 1	Mean 2	Mean Diff.	SE of diff.	N1	N2	t	DF	95.00% CI of diff.	Adjusted P Value
**Pert1$_R$**	Young										
	Base vs. Pre	0.6217	0.6173	0.004363	0.005348	30	30	0.8158	29	-0.01166 to 0.02038	>0.9999
	Base vs. Post1	0.6217	0.8191	-0.1974	0.01943	30	30	10.16	29	-0.2557 to -0.1392	<0.0001
	Base vs. Post2	0.6217	0.3928	0.2289	0.02759	30	30	8.294	29	0.1462 to 0.3115	<0.0001
	Base vs. Post3	0.6217	0.6951	-0.07339	0.02688	30	30	2.731	29	-0.1539 to 0.007129	0.0958
	Base vs. Post4	0.6217	0.7456	-0.124	0.01765	30	30	7.024	29	-0.1768 to -0.07109	<0.0001
	Base vs. Post5	0.6217	0.6405	-0.01884	0.01662	30	30	1.133	29	-0.06864 to 0.03097	>0.9999
	Base vs. Post6	0.6217	0.626	-0.004297	0.01361	30	30	0.3157	29	-0.04508 to 0.03649	>0.9999
	Base vs. Post7	0.6217	0.6061	0.01557	0.00955	30	30	1.631	29	-0.01304 to 0.04418	>0.9999
	Base vs. Post8	0.6217	0.6114	0.01028	0.01082	30	30	0.9504	29	-0.02213 to 0.04269	>0.9999
	Older										
	Base vs. Pre	0.5942	0.5942	-9.535E-06	0.005839	27	27	0.0016	26	-0.01767 to 0.01765	>0.9999
	Base vs. Post1	0.5942	0.6314	-0.03724	0.01465	27	27	2.542	26	-0.08154 to 0.007056	0.1559
	Base vs. Post2	0.5942	0.2209	0.3733	0.03143	27	27	11.88	26	0.2783 to 0.4683	<0.0001
	Base vs. Post3	0.5942	0.5272	0.06696	0.02828	27	27	2.368	26	-0.01856 to 0.1525	0.2307
	Base vs. Post4	0.5942	0.6282	-0.03402	0.0273	27	27	1.246	26	-0.1166 to 0.04853	>0.9999
	Base vs. Post5	0.5942	0.6302	-0.03604	0.02071	27	27	1.741	26	-0.09866 to 0.02657	0.8421
	Base vs. Post6	0.5942	0.644	-0.04984	0.0135	27	27	3.692	26	-0.09065 to -0.009022	0.0093
	Base vs. Post7	0.5942	0.6001	-0.0059	0.01252	27	27	0.4712	26	-0.04376 to 0.03196	>0.9999
	Base vs. Post8	0.5942	0.6029	-0.00868	0.01323	27	27	0.656	26	-0.04869 to 0.03133	>0.9999
**Pert2$_L$**	Young										
	Base vs. Pre	0.6201	0.6159	0.004229	0.00424	30	30	0.9974	29	-0.008474 to 0.01693	>0.9999
	Base vs. Post1	0.6201	0.8288	-0.2087	0.01824	30	30	11.44	29	-0.2634 to -0.1541	<0.0001
	Base vs. Post2	0.6201	0.4474	0.1727	0.02634	30	30	6.556	29	0.09378 to 0.2516	<0.0001
	Base vs. Post3	0.6201	0.6679	-0.04785	0.02408	30	30	1.987	29	-0.1200 to 0.02430	0.508
	Base vs. Post4	0.6201	0.7533	-0.1333	0.01764	30	30	7.553	29	-0.1861 to -0.08040	<0.0001
	Base vs. Post5	0.6201	0.6604	-0.04032	0.01577	30	30	2.558	29	-0.08755 to 0.006911	0.1443
	Base vs. Post6	0.6201	0.6277	-0.007645	0.01022	30	30	0.7478	29	-0.03827 to 0.02298	>0.9999
	Base vs. Post7	0.6201	0.6007	0.0194	0.009114	30	30	2.128	29	-0.007906 to 0.04670	0.3773
	Base vs. Post8	0.6201	0.6173	0.002745	0.007312	30	30	0.3754	29	-0.01916 to 0.02465	>0.9999
	Older										
	Base vs. Pre	0.5893	0.5877	0.001586	0.006807	28	28	0.233	27	-0.01893 to 0.02210	>0.9999
	Base vs. Post1	0.5893	0.672	-0.08275	0.02284	28	28	3.623	27	-0.1516 to -0.01392	0.0107
	Base vs. Post2	0.5893	0.2663	0.3229	0.0189	28	28	17.08	27	0.2660 to 0.3799	<0.0001
	Base vs. Post3	0.5893	0.5299	0.05935	0.01948	28	28	3.046	27	0.0006364 to 0.1181	0.0461
	Base vs. Post4	0.5893	0.6351	-0.04588	0.02789	28	28	1.645	27	-0.1299 to 0.03816	>0.9999

	Base vs. Post5	0.5893	0.637	-0.04778	0.01887	28	28	2.533	27	-0.1046 to 0.009073	0.1569
	Base vs. Post6	0.5893	0.6674	-0.07809	0.01426	28	28	5.475	27	-0.1211 to -0.03511	<0.0001
	Base vs. Post7	0.5893	0.6177	-0.02842	0.01173	28	28	2.423	27	-0.06376 to 0.006925	0.2013
	Base vs. Post8	0.5893	0.6032	-0.01394	0.00787	28	28	1.771	27	-0.03765 to 0.009781	0.7908
**Pert9$_L$**	Young										
	Base vs. Pre	0.6212	0.6211	0.0001787	0.005884	30	30	0.0304	29	-0.01745 to 0.01781	>0.9999
	Base vs. Post1	0.6212	0.812	-0.1907	0.02334	30	30	8.171	29	-0.2607 to -0.1208	<0.0001
	Base vs. Post2	0.6212	0.5942	0.02708	0.01529	30	30	1.771	29	-0.01873 to 0.07289	0.784
	Base vs. Post3	0.6212	0.6296	-0.008359	0.01927	30	30	0.4337	29	-0.06610 to 0.04938	>0.9999
	Base vs. Post4	0.6212	0.6864	-0.06513	0.01207	30	30	5.397	29	-0.1013 to -0.02897	<0.0001
	Base vs. Post5	0.6212	0.6354	-0.0142	0.009939	30	30	1.429	29	-0.04398 to 0.01557	>0.9999
	Base vs. Post6	0.6212	0.6274	-0.006144	0.007752	30	30	0.7926	29	-0.02937 to 0.01708	>0.9999
	Base vs. Post7	0.6212	0.6069	0.01438	0.007794	30	30	1.845	29	-0.008973 to 0.03772	0.678
	Base vs. Post8	0.6212	0.6117	0.009525	0.007469	30	30	1.275	29	-0.01285 to 0.03190	>0.9999
	Older										
	Base vs. Pre	0.5874	0.5908	-0.003338	0.006542	28	28	0.5102	27	-0.02305 to 0.01638	>0.9999
	Base vs. Post1	0.5874	0.7392	-0.1518	0.02003	28	28	7.578	27	-0.2121 to -0.09141	<0.0001
	Base vs. Post2	0.5874	0.4984	0.08902	0.02295	28	28	3.879	27	0.01986 to 0.1582	0.0055
	Base vs. Post3	0.5874	0.5552	0.0322	0.01658	28	28	1.942	27	-0.01778 to 0.08217	0.5641
	Base vs. Post4	0.5874	0.6293	-0.04184	0.01663	28	28	2.517	27	-0.09195 to 0.008264	0.1628
	Base vs. Post5	0.5874	0.6005	-0.01306	0.01649	28	28	0.792	27	-0.06276 to 0.03664	>0.9999
	Base vs. Post6	0.5874	0.624	-0.03656	0.01415	28	28	2.583	27	-0.07921 to 0.006095	0.1398
	Base vs. Post7	0.5874	0.5844	0.002988	0.009635	28	28	0.3102	27	-0.02605 to 0.03203	>0.9999
	Base vs. Post8	0.5874	0.597	-0.009543	0.009634	28	28	0.9906	27	-0.03858 to 0.01949	>0.9999

eTable 6: Bonferroni's multiple comparison test results comparing the young and older adults in Pert1$_R$, Pert2$_L$ and Pert9$_L$ for X$_{CoM}$ [m]

Perturbation	Test details	Mean 1	Mean 2	Mean Diff.	SE of diff.	N1	N2	t	DF	95.00% CI of diff.	Adjusted P Value
**Pert1$_R$**	Young - Older										
	Base	0.6217	0.5942	0.02748	0.01341	30	27	2.049	40.77	-0.01232 to 0.06728	0.4692
	Pre	0.6173	0.5942	0.02311	0.01418	30	27	1.629	47	-0.01867 to 0.06488	>0.9999
	Post1	0.8191	0.6314	0.1877	0.02587	30	27	7.255	53.23	0.1119 to 0.2634	<0.0001
	Post2	0.3928	0.2209	0.1719	0.04256	30	27	4.04	52.7	0.04722 to 0.2966	0.0017
	Post3	0.6951	0.5272	0.1678	0.04267	30	27	3.933	52.05	0.04271 to 0.2929	0.0025
	Post4	0.7456	0.6282	0.1174	0.03759	30	27	3.123	44.95	0.006426 to 0.2284	0.0313
	Post5	0.6405	0.6302	0.01027	0.03197	30	27	0.3213	47.22	-0.08387 to 0.1044	>0.9999
	Post6	0.626	0.644	-0.01806	0.02611	30	27	0.6916	52.83	-0.09457 to 0.05845	>0.9999
	Post7	0.6061	0.6001	0.006003	0.02362	30	27	0.2541	44.82	-0.06374 to 0.07575	>0.9999
	Post8	0.6114	0.6029	0.008517	0.02263	30	27	0.3763	51.81	-0.05784 to 0.07487	>0.9999
**Pert2$_L$**	Young - Older										
	Base	0.6201	0.5893	0.03083	0.01398	30	28	2.205	43.42	-0.01052 to 0.07218	0.328
	Pre	0.6159	0.5877	0.02819	0.01531	30	28	1.841	46.81	-0.01692 to 0.07329	0.7196
	Post1	0.8288	0.672	0.1568	0.0318	30	28	4.93	52.57	0.06360 to 0.2500	<0.0001
	Post2	0.4474	0.2663	0.1811	0.03541	30	28	5.112	54.18	0.07741 to 0.2847	<0.0001
	Post3	0.6679	0.5299	0.138	0.03578	30	28	3.858	54.81	0.03337 to 0.2427	0.003
	Post4	0.7533	0.6351	0.1182	0.03644	30	28	3.244	47.29	0.01090 to 0.2255	0.0217
	Post5	0.6604	0.637	0.02337	0.02945	30	28	0.7933	54.4	-0.06282 to 0.1096	>0.9999
	Post6	0.6277	0.6674	-0.03962	0.02214	30	28	1.79	51.7	-0.1045 to 0.02530	0.7934
	Post7	0.6007	0.6177	-0.01699	0.01807	30	28	0.9402	54.36	-0.06986 to 0.03588	>0.9999
	Post8	0.6173	0.6032	0.01415	0.01601	30	28	0.8835	46.49	-0.03304 to 0.06134	>0.9999
**Pert9$_L$**	Young - Older										
	Base	0.6212	0.5874	0.03381	0.01298	30	28	2.605	43.69	-0.004570 to 0.07218	0.1253
	Pre	0.6211	0.5908	0.03029	0.01509	30	28	2.007	48.84	-0.01408 to 0.07466	0.5028
	Post1	0.812	0.7392	0.07278	0.03309	30	28	2.199	55.97	-0.02394 to 0.1695	0.3202
	Post2	0.5942	0.4984	0.09575	0.03315	30	28	2.888	45.77	-0.002033 to 0.1935	0.059
	Post3	0.6296	0.5552	0.07436	0.02908	30	28	2.557	55.99	-0.01061 to 0.1593	0.1328
	Post4	0.6864	0.6293	0.05709	0.02222	30	28	2.57	54.15	-0.007934 to 0.1221	0.1297
	Post5	0.6354	0.6005	0.03495	0.02058	30	28	1.698	49.95	-0.02549 to 0.09538	0.9564
	Post6	0.6274	0.624	0.003393	0.02126	30	28	0.1595	46.66	-0.05927 to 0.06605	>0.9999
	Post7	0.6069	0.5844	0.02242	0.0179	30	28	1.252	50.23	-0.03014 to 0.07498	>0.9999
	Post8	0.6117	0.597	0.01474	0.01775	30	28	0.8302	43.64	-0.03776 to 0.06723	>0.9999

eTable 7: Bonferroni's multiple comparison test results comparing recovery steps to baseline in Pert1$_R$, Pert2$_L$ and Pert9$_L$ for MoS$_{ML}$ [m] in the young and older adults

Perturbation	Test details	Mean 1	Mean 2	Mean Diff.	SE of diff.	N1	N2	t	DF	95.00% CI of diff.	Adjusted P Value
Pert1$_R$	Young										
	Base vs. Pre	0.00993	0.01381	-0.003881	0.002857	30	30	1.358	29	-0.01244 to 0.004678	>0.9999
	Base vs. Post1	0.00993	-0.01824	0.02817	0.006462	30	30	4.359	29	0.008809 to 0.04753	0.0013
	Base vs. Post2	0.00993	0.05714	-0.04721	0.007052	30	30	6.694	29	-0.06834 to -0.02608	<0.0001
	Base vs. Post3	0.00993	0.02722	-0.01729	0.006142	30	30	2.815	29	-0.03569 to 0.001108	0.078
	Base vs. Post4	0.00993	0.01635	-0.006418	0.004151	30	30	1.546	29	-0.01885 to 0.006019	>0.9999
	Base vs. Post5	0.00993	0.01654	-0.006613	0.003173	30	30	2.084	29	-0.01612 to 0.002892	0.4144
	Base vs. Post6	0.00993	0.01908	-0.009145	0.005265	30	30	1.737	29	-0.02492 to 0.006629	0.8372
	Base vs. Post7	0.00993	0.01768	-0.007747	0.002813	30	30	2.754	29	-0.01617 to 0.0006799	0.0905
	Base vs. Post8	0.00993	0.02129	-0.01136	0.003613	30	30	3.145	29	-0.02219 to -0.0005390	0.0343
	Older										
	Base vs. Pre	0.01644	0.02095	-0.004507	0.00331	27	27	1.362	26	-0.01452 to 0.005503	>0.9999
	Base vs. Post1	0.01644	0.005855	0.01058	0.008328	27	27	1.271	26	-0.01460 to 0.03576	>0.9999
	Base vs. Post2	0.01644	0.0454	-0.02897	0.00871	27	27	3.325	26	-0.05530 to -0.002628	0.0237
	Base vs. Post3	0.01644	0.03473	-0.01829	0.009911	27	27	1.845	26	-0.04826 to 0.01168	0.6876
	Base vs. Post4	0.01644	0.04115	-0.02471	0.005466	27	27	4.521	26	-0.04124 to -0.008185	0.0011
	Base vs. Post5	0.01644	0.02488	-0.00844	0.00469	27	27	1.799	26	-0.02262 to 0.005742	0.7521
	Base vs. Post6	0.01644	0.03278	-0.01634	0.004258	27	27	3.837	26	-0.02922 to -0.003464	0.0064
	Base vs. Post7	0.01644	0.02323	-0.006792	0.004249	27	27	1.598	26	-0.01964 to 0.006056	>0.9999
	Base vs. Post8	0.01644	0.02829	-0.01185	0.004343	27	27	2.729	26	-0.02499 to 0.001278	0.1011
Pert2$_L$	Young										
	Base vs. Pre	0.01463	0.01294	0.001691	0.00324	30	30	0.5218	29	-0.008015 to 0.01140	>0.9999
	Base vs. Post1	0.01463	-0.01619	0.03082	0.009414	30	30	3.274	29	0.002616 to 0.05902	0.0247
	Base vs. Post2	0.01463	0.06054	-0.0459	0.006201	30	30	7.402	29	-0.06448 to -0.02732	<0.0001
	Base vs. Post3	0.01463	0.03341	-0.01877	0.004804	30	30	3.908	29	-0.03317 to -0.004380	0.0046
	Base vs. Post4	0.01463	0.0199	-0.00527	0.003388	30	30	1.555	29	-0.01542 to 0.004880	>0.9999
	Base vs. Post5	0.01463	0.01923	-0.004594	0.003308	30	30	1.389	29	-0.01450 to 0.005316	>0.9999
	Base vs. Post6	0.01463	0.02158	-0.006944	0.003387	30	30	2.05	29	-0.01709 to 0.003203	0.4453
	Base vs. Post7	0.01463	0.02254	-0.007906	0.002615	30	30	3.024	29	-0.01574 to -7.218e-005	0.0467
	Base vs. Post8	0.01463	0.01893	-0.004295	0.002056	30	30	2.089	29	-0.01045 to 0.001864	0.4104
	Older										
	Base vs. Pre	0.01851	0.01986	-0.00135	0.002821	28	28	0.4788	27	-0.009851 to 0.007150	>0.9999
	Base vs. Post1	0.01851	0.003911	0.0146	0.00723	28	28	2.019	27	-0.007189 to 0.03639	0.4813
	Base vs. Post2	0.01851	0.04397	-0.02545	0.00709	28	28	3.59	27	-0.04682 to -0.004086	0.0117
	Base vs. Post3	0.01851	0.04124	-0.02273	0.005476	28	28	4.15	27	-0.03923 to -0.006223	0.0027
	Base vs. Post4	0.01851	0.02997	-0.01146	0.005122	28	28	2.237	27	-0.02690 to 0.003977	0.3035

	Base vs. Post5	0.01851	0.03564	-0.01712	0.005769	28	28	2.969	27	-0.03451 to 0.0002602	0.0558
	Base vs. Post6	0.01851	0.02377	-0.005261	0.004111	28	28	1.28	27	-0.01765 to 0.007128	>0.9999
	Base vs. Post7	0.01851	0.02978	-0.01126	0.004092	28	28	2.753	27	-0.02360 to 0.001068	0.0939
	Base vs. Post8	0.01851	0.02537	-0.006858	0.003129	28	28	2.192	27	-0.01629 to 0.002572	0.3348
**$Pert9_L$**	Young										
	Base vs. Pre	0.01167	0.01541	-0.003741	0.002662	30	30	1.406	29	-0.01172 to 0.004233	>0.9999
	Base vs. Post1	0.01167	0.01429	-0.002621	0.006735	30	30	0.3891	29	-0.02280 to 0.01756	>0.9999
	Base vs. Post2	0.01167	0.04776	-0.03609	0.005783	30	30	6.242	29	-0.05342 to -0.01877	<0.0001
	Base vs. Post3	0.01167	0.02938	-0.01772	0.004635	30	30	3.822	29	-0.03160 to -0.003830	0.0058
	Base vs. Post4	0.01167	0.01602	-0.004356	0.003774	30	30	1.154	29	-0.01566 to 0.006952	>0.9999
	Base vs. Post5	0.01167	0.01403	-0.002361	0.003278	30	30	0.7201	29	-0.01218 to 0.007460	>0.9999
	Base vs. Post6	0.01167	0.008163	0.003503	0.002902	30	30	1.207	29	-0.005190 to 0.01220	>0.9999
	Base vs. Post7	0.01167	0.01456	-0.002889	0.003158	30	30	0.9149	29	-0.01235 to 0.006571	>0.9999
	Base vs. Post8	0.01167	0.009144	0.002523	0.002191	30	30	1.151	29	-0.004042 to 0.009087	>0.9999
	Older										
	Base vs. Pre	0.02119	0.01881	0.002382	0.003569	28	28	0.6672	27	-0.008375 to 0.01314	>0.9999
	Base vs. Post1	0.02119	-0.001606	0.0228	0.008087	28	28	2.819	27	-0.001575 to 0.04717	0.0802
	Base vs. Post2	0.02119	0.04653	-0.02534	0.005958	28	28	4.253	27	-0.04330 to -0.007386	0.002
	Base vs. Post3	0.02119	0.04963	-0.02844	0.00501	28	28	5.677	27	-0.04354 to -0.01334	<0.0001
	Base vs. Post4	0.02119	0.0268	-0.005611	0.005691	28	28	0.986	27	-0.02276 to 0.01154	>0.9999
	Base vs. Post5	0.02119	0.03289	-0.01169	0.003262	28	28	3.585	27	-0.02152 to -0.001864	0.0118
	Base vs. Post6	0.02119	0.02511	-0.00392	0.00281	28	28	1.395	27	-0.01239 to 0.004547	>0.9999
	Base vs. Post7	0.02119	0.02618	-0.004989	0.003031	28	28	1.646	27	-0.01412 to 0.004146	>0.9999
	Base vs. Post8	0.02119	0.01847	0.002723	0.00302	28	28	0.9016	27	-0.006379 to 0.01183	>0.9999

eTable 8: Bonferroni's multiple comparison test results comparing the young and older adults in $Pert1_R$, $Pert2_L$ and $Pert9_L$ for MoS_{ML} [m]

Perturbation	Test details	Mean 1	Mean 2	Mean Diff.	SE of diff.	N1	N2	t	DF	95.00% CI of diff.	Adjusted P Value
**$Pert1_R$**	Young - Older										
	Base	0.00993	0.01644	-0.006509	0.003998	30	27	1.628	52.73	-0.01822 to 0.005206	>0.9999
	Pre	0.01381	0.02095	-0.007135	0.005738	30	27	1.243	53.19	-0.02394 to 0.009672	>0.9999
	Post1	-0.01824	0.005855	-0.02409	0.009986	30	27	2.413	51.33	-0.05339 to 0.005199	0.1944
	Post2	0.05714	0.0454	0.01174	0.01095	30	27	1.072	49.5	-0.02044 to 0.04391	>0.9999
	Post3	0.02722	0.03473	-0.007506	0.01242	30	27	0.6042	40.22	-0.04440 to 0.02939	>0.9999
	Post4	0.01635	0.04115	-0.0248	0.007797	30	27	3.181	50.04	-0.04770 to -0.001907	0.0252
	Post5	0.01654	0.02488	-0.008335	0.006806	30	27	1.225	52.35	-0.02828 to 0.01161	>0.9999
	Post6	0.01908	0.03278	-0.0137	0.008015	30	27	1.71	53.68	-0.03717 to 0.009762	0.9308
	Post7	0.01768	0.02323	-0.005553	0.006228	30	27	0.8916	54.22	-0.02378 to 0.01267	>0.9999
	Post8	0.02129	0.02829	-0.007001	0.006483	30	27	1.08	51.49	-0.02602 to 0.01202	>0.9999
**$Pert2_L$**	Young - Older										
	Base	0.01463	0.01851	-0.003877	0.004182	30	28	0.9271	55.37	-0.01610 to 0.008350	>0.9999
	Pre	0.01294	0.01986	-0.006918	0.005662	30	28	1.222	55.99	-0.02346 to 0.009629	>0.9999
	Post1	-0.01619	0.003911	-0.0201	0.01263	30	28	1.591	50.78	-0.05717 to 0.01698	>0.9999
	Post2	0.06054	0.04397	0.01657	0.01045	30	28	1.586	53.62	-0.01402 to 0.04716	>0.9999
	Post3	0.03341	0.04124	-0.007828	0.007677	30	28	1.02	48.24	-0.03041 to 0.01476	>0.9999
	Post4	0.0199	0.02997	-0.01007	0.006901	30	28	1.459	50.48	-0.03033 to 0.01019	>0.9999
	Post5	0.01923	0.03564	-0.01641	0.00749	30	28	2.19	42.79	-0.03858 to 0.005764	0.3399
	Post6	0.02158	0.02377	-0.002194	0.006056	30	28	0.3623	54.83	-0.01991 to 0.01552	>0.9999
	Post7	0.02254	0.02978	-0.007235	0.006073	30	28	1.191	47.56	-0.02511 to 0.01064	>0.9999
	Post8	0.01893	0.02537	-0.00644	0.005667	30	28	1.136	53.54	-0.02303 to 0.01015	>0.9999
**$Pert9_L$**	Young - Older										
	Base	0.01167	0.02119	-0.009525	0.0047	30	28	2.027	53.32	-0.02329 to 0.004239	0.4772
	Pre	0.01541	0.01881	-0.003402	0.005727	30	28	0.594	55.22	-0.02015 to 0.01335	>0.9999
	Post1	0.01429	-0.001606	0.01589	0.01056	30	28	1.506	55.98	-0.01496 to 0.04674	>0.9999
	Post2	0.04776	0.04653	0.001227	0.00871	30	28	0.1409	53.5	-0.02428 to 0.02673	>0.9999
	Post3	0.02938	0.04963	-0.02025	0.006971	30	28	2.905	55.99	-0.04062 to 0.0001226	0.0525
	Post4	0.01602	0.0268	-0.01078	0.007162	30	28	1.505	42.74	-0.03198 to 0.01042	>0.9999
	Post5	0.01403	0.03289	-0.01886	0.005902	30	28	3.195	52.51	-0.03615 to -0.001563	0.0236
	Post6	0.00816	0.02511	-0.01695	0.005176	30	28	3.275	54.59	-0.03209 to -0.001806	0.0184
	Post7	0.01456	0.02618	-0.01162	0.005771	30	28	2.014	52.19	-0.02854 to 0.005293	0.4915
	Post8	0.00914	0.01847	-0.009325	0.005245	30	28	1.778	46.84	-0.02478 to 0.006128	0.8194

eTable 9: Bonferroni's multiple comparison test results comparing recovery steps to baseline in Pert1$_R$ and Pert10$_R$ for MoS$_{AP}$ [m] in the older adults

Test details	Mean 1	Mean 2	Mean Diff.	SE of diff.	N1	N2	t	DF	95.00% CI of diff.	Adjusted P Value
Pert1$_R$										
Base vs. Pre	0.0496	0.04576	0.003836	0.003399	27	27	1.128	26	-0.006443 to 0.01411	>0.9999
Base vs. Post1	0.0496	0.1117	-0.06213	0.01244	27	27	4.996	26	-0.09973 to -0.02452	0.0003
Base vs. Post2	0.0496	0.02267	0.02693	0.01389	27	27	1.939	26	-0.01506 to 0.06892	0.5709
Base vs. Post3	0.0496	-0.0007875	0.05038	0.01063	27	27	4.739	26	0.01824 to 0.08253	0.0006
Base vs. Post4	0.0496	-0.02787	0.07746	0.01084	27	27	7.143	26	0.04467 to 0.1103	<0.0001
Base vs. Post5	0.0496	-0.009746	0.05934	0.01015	27	27	5.846	26	0.02865 to 0.09004	<0.0001
Base vs. Post6	0.0496	0.004204	0.04539	0.007151	27	27	6.348	26	0.02377 to 0.06702	<0.0001
Base vs. Post7	0.0496	0.01565	0.03395	0.007473	27	27	4.542	26	0.01135 to 0.05654	0.001
Base vs. Post8	0.0496	0.02613	0.02347	0.0077	27	27	3.048	26	0.0001856 to 0.04675	0.0471
Pert10$_R$										
Base vs. Pre	0.04955	0.04616	0.003387	0.004206	27	27	0.8053	26	-0.009330 to 0.01610	>0.9999
Base vs. Post1	0.04955	0.01955	0.02999	0.01472	27	27	2.038	26	-0.01452 to 0.07450	0.467
Base vs. Post2	0.04955	-0.03525	0.08479	0.01148	27	27	7.388	26	0.05009 to 0.1195	<0.0001
Base vs. Post3	0.04955	-0.0292	0.07875	0.01147	27	27	6.868	26	0.04408 to 0.1134	<0.0001
Base vs. Post4	0.04955	-0.01534	0.06489	0.00867	27	27	7.484	26	0.03867 to 0.09110	<0.0001
Base vs. Post5	0.04955	0.01999	0.02956	0.007947	27	27	3.719	26	0.005526 to 0.05359	0.0087
Base vs. Post6	0.04955	0.03602	0.01353	0.008877	27	27	1.524	26	-0.01331 to 0.04037	>0.9999
Base vs. Post7	0.04955	0.03715	0.01239	0.005516	27	27	2.247	26	-0.004286 to 0.02907	0.3004
Base vs. Post8	0.04955	0.04975	-0.0002044	0.00441	27	27	0.0463	26	-0.01354 to 0.01313	>0.9999

eTable 10: Bonferroni's multiple comparison test results comparing Pert1$_R$ to Pert10$_R$ for MoS$_{AP}$ [m] in the older adults

Test details	Mean 1	Mean 2	Mean Diff.	SE of diff.	N1	N2	t	DF	95.00% CI of diff.	Adjusted P Value
Pert1$_R$ - Pert10$_R$										
Base	0.0496	0.04955	5.044E-05	0.001873	27	27	0.0269	26	-0.005693 to 0.005794	>0.9999
Pre	0.04576	0.04616	-0.0003983	0.003603	27	27	0.1106	26	-0.01145 to 0.01065	>0.9999
Post1	0.1117	0.01955	0.09217	0.01073	27	27	8.586	26	0.05925 to 0.1251	<0.0001
Post2	0.02267	-0.03525	0.05792	0.01635	27	27	3.542	26	0.007766 to 0.1081	0.0152
Post3	-0.0008	-0.0292	0.02842	0.01276	27	27	2.227	26	-0.01072 to 0.06756	0.3485
Post4	-0.0279	-0.01534	-0.01252	0.012	27	27	1.043	26	-0.04934 to 0.02429	>0.9999
Post5	-0.0097	0.01999	-0.02974	0.01126	27	27	2.642	26	-0.06426 to 0.004782	0.1377
Post6	0.0042	0.03602	-0.03181	0.01121	27	27	2.838	26	-0.06619 to 0.002567	0.0869
Post7	0.01565	0.03715	-0.0215	0.008091	27	27	2.658	26	-0.04632 to 0.003310	0.1327
Post8	0.02613	0.04975	-0.02362	0.006687	27	27	3.533	26	-0.04413 to -0.003115	0.0156

eTable 11: Bonferroni's multiple comparison test results comparing recovery steps to baseline in Pert1R and Pert10R for BoS [m] in the older adults

Test details	Mean 1	Mean 2	Mean Diff.	SE of diff.	N1	N2	t	DF	95.00% CI of diff.	Adjusted P Value
Pert1R										
Base vs. Pre	0.6438	0.64	0.003826	0.004034	27	27	0.9484	26	-0.008373 to 0.01603	>0.9999
Base vs. Post1	0.6438	0.7432	-0.09937	0.01022	27	27	9.723	26	-0.1303 to -0.06847	<0.0001
Base vs. Post2	0.6438	0.2436	0.4002	0.03247	27	27	12.33	26	0.3020 to 0.4984	<0.0001
Base vs. Post3	0.6438	0.5265	0.1173	0.02718	27	27	4.318	26	0.03516 to 0.1995	0.0018
Base vs. Post4	0.6438	0.6003	0.04344	0.02796	27	27	1.554	26	-0.04109 to 0.1280	>0.9999
Base vs. Post5	0.6438	0.6205	0.0233	0.01868	27	27	1.248	26	-0.03317 to 0.07977	>0.9999
Base vs. Post6	0.6438	0.6482	-0.004443	0.01213	27	27	0.3664	26	-0.04111 to 0.03222	>0.9999
Base vs. Post7	0.6438	0.6157	0.02805	0.01354	27	27	2.071	26	-0.01290 to 0.06899	0.4355
Base vs. Post8	0.6438	0.629	0.01479	0.01053	27	27	1.404	26	-0.01705 to 0.04663	>0.9999
Pert10R										
Base vs. Pre	0.6519	0.6503	0.001648	0.005721	27	27	0.288	26	-0.01565 to 0.01895	>0.9999
Base vs. Post1	0.6519	0.7293	-0.07739	0.01508	27	27	5.133	26	-0.1230 to -0.03180	0.0002
Base vs. Post2	0.6519	0.3814	0.2705	0.03636	27	27	7.441	26	0.1606 to 0.3805	<0.0001
Base vs. Post3	0.6519	0.5905	0.06141	0.01585	27	27	3.873	26	0.01347 to 0.1093	0.0059
Base vs. Post4	0.6519	0.607	0.04488	0.02076	27	27	2.161	26	-0.01791 to 0.1077	0.3605
Base vs. Post5	0.6519	0.636	0.01587	0.01416	27	27	1.121	26	-0.02695 to 0.05868	>0.9999
Base vs. Post6	0.6519	0.646	0.005856	0.009091	27	27	0.6442	26	-0.02163 to 0.03334	>0.9999
Base vs. Post7	0.6519	0.6313	0.02063	0.01164	27	27	1.773	26	-0.01455 to 0.05582	0.7912
Base vs. Post8	0.6519	0.6456	0.006321	0.006719	27	27	0.9407	26	-0.01400 to 0.02664	>0.9999

eTable 12: Bonferroni's multiple comparison test results comparing Pert1R to Pert10R for BoS [m] in the older adults

Test details	Mean 1	Mean 2	Mean Diff.	SE of diff.	N1	N2	t	DF	95.00% CI of diff.	Adjusted P Value
Pert1R - Pert10R										
Base	0.6438	0.6519	-0.008115	0.004207	27	27	1.929	26	-0.02102 to 0.004789	0.6476
Pre	0.64	0.6503	-0.01029	0.006064	27	27	1.697	26	-0.02889 to 0.008306	>0.9999
Post1	0.7432	0.7293	0.01386	0.0113	27	27	1.227	26	-0.02078 to 0.04850	>0.9999
Post2	0.2436	0.3814	-0.1378	0.04262	27	27	3.233	26	-0.2685 to -0.007088	0.0332
Post3	0.5265	0.5905	-0.06405	0.02388	27	27	2.682	26	-0.1373 to 0.009181	0.1253
Post4	0.6003	0.607	-0.00668	0.02648	27	27	0.2523	26	-0.08788 to 0.07452	>0.9999
Post5	0.6205	0.636	-0.01555	0.01339	27	27	1.161	26	-0.05663 to 0.02553	>0.9999
Post6	0.6482	0.646	0.002185	0.01249	27	27	0.175	26	-0.03611 to 0.04048	>0.9999
Post7	0.6157	0.6313	-0.01553	0.01433	27	27	1.084	26	-0.05948 to 0.02842	>0.9999
Post8	0.629	0.6456	-0.01658	0.01148	27	27	1.444	26	-0.05180 to 0.01863	>0.9999

eTable 13: Bonferroni's multiple comparison test results comparing recovery steps to baseline in Pert1$_R$ and Pert10$_R$ for X_{CoM} [m] in the older adults

Test details	Mean 1	Mean 2	Mean Diff.	SE of diff.	N1	N2	t	DF	95.00% CI of diff.	Adjusted P Value
Pert1$_R$										
Base vs. Pre	0.5942	0.5942	-9.535E-06	0.005839	27	27	0.0016	26	-0.01767 to 0.01765	>0.9999
Base vs. Post1	0.5942	0.6314	-0.03724	0.01465	27	27	2.542	26	-0.08154 to 0.007056	0.1559
Base vs. Post2	0.5942	0.2209	0.3733	0.03143	27	27	11.88	26	0.2783 to 0.4683	<0.0001
Base vs. Post3	0.5942	0.5272	0.06696	0.02828	27	27	2.368	26	-0.01856 to 0.1525	0.2307
Base vs. Post4	0.5942	0.6282	-0.03402	0.0273	27	27	1.246	26	-0.1166 to 0.04853	>0.9999
Base vs. Post5	0.5942	0.6302	-0.03604	0.02071	27	27	1.741	26	-0.09866 to 0.02657	0.8421
Base vs. Post6	0.5942	0.644	-0.04984	0.0135	27	27	3.692	26	-0.09065 to -0.009022	0.0093
Base vs. Post7	0.5942	0.6001	-0.0059	0.01252	27	27	0.4712	26	-0.04376 to 0.03196	>0.9999
Base vs. Post8	0.5942	0.6029	-0.00868	0.01323	27	27	0.656	26	-0.04869 to 0.03133	>0.9999
Pert10$_R$										
Base vs. Pre	0.6024	0.6041	-0.001739	0.007199	27	27	0.2416	26	-0.02351 to 0.02003	>0.9999
Base vs. Post1	0.6024	0.7097	-0.1074	0.01619	27	27	6.635	26	-0.1563 to -0.05844	<0.0001
Base vs. Post2	0.6024	0.4166	0.1857	0.0339	27	27	5.479	26	0.08324 to 0.2882	<0.0001
Base vs. Post3	0.6024	0.6197	-0.01734	0.02256	27	27	0.7686	26	-0.08557 to 0.05088	>0.9999
Base vs. Post4	0.6024	0.6224	-0.02001	0.01829	27	27	1.094	26	-0.07532 to 0.03530	>0.9999
Base vs. Post5	0.6024	0.616	-0.01369	0.01932	27	27	0.7087	26	-0.07210 to 0.04472	>0.9999
Base vs. Post6	0.6024	0.61	-0.007673	0.01552	27	27	0.4944	26	-0.05460 to 0.03925	>0.9999
Base vs. Post7	0.6024	0.5941	0.00824	0.01461	27	27	0.5641	26	-0.03593 to 0.05241	>0.9999
Base vs. Post8	0.6024	0.5958	0.006525	0.008906	27	27	0.7327	26	-0.02040 to 0.03345	>0.9999

eTable 14: Bonferroni's multiple comparison test results comparing Pert1$_R$ to Pert10$_R$ for X_{CoM} [m] in the older adults

Test details	Mean 1	Mean 2	Mean Diff.	SE of diff.	N1	N2	t	DF	95.00% CI of diff.	Adjusted P Value
Pert1$_R$ - Pert10$_R$										
Base	0.5942	0.6024	-0.008165	0.004169	27	27	1.959	26	-0.02095 to 0.004620	0.6095
Pre	0.5942	0.6041	-0.009895	0.007249	27	27	1.365	26	-0.03213 to 0.01234	>0.9999
Post1	0.6314	0.7097	-0.07831	0.01123	27	27	6.976	26	-0.1127 to -0.04388	<0.0001
Post2	0.2209	0.4166	-0.1957	0.03769	27	27	5.193	26	-0.3113 to -0.08013	0.0002
Post3	0.5272	0.6197	-0.09246	0.02854	27	27	3.24	26	-0.1800 to -0.004932	0.0326
Post4	0.6282	0.6224	0.005845	0.02627	27	27	0.2225	26	-0.07471 to 0.08640	>0.9999
Post5	0.6302	0.616	0.01419	0.01757	27	27	0.8074	26	-0.03970 to 0.06808	>0.9999
Post6	0.644	0.61	0.034	0.01895	27	27	1.794	26	-0.02414 to 0.09213	0.8452
Post7	0.6001	0.5941	0.005975	0.01438	27	27	0.4156	26	-0.03812 to 0.05007	>0.9999
Post8	0.6029	0.5958	0.00704	0.01329	27	27	0.5298	26	-0.03371 to 0.04779	>0.9999

eTable 15: Bonferroni's multiple comparison test results comparing recovery steps to baseline in $Pert1_R$ and $Pert10_R$ for MoS_{ML} [m] in the older adults

Test details	Mean 1	Mean 2	Mean Diff.	SE of diff.	N1	N2	t	DF	95.00% CI of diff.	Adjusted P Value
$Pert1_R$										
Base vs. Pre	0.01644	0.02095	-0.004507	0.003311	27	27	1.361	26	-0.01452 to 0.005503	>0.9999
Base vs. Post1	0.01644	0.005856	0.01058	0.008328	27	27	1.271	26	-0.01460 to 0.03576	>0.9999
Base vs. Post2	0.01644	0.0454	-0.02897	0.00871	27	27	3.326	26	-0.05530 to -0.002629	0.0237
Base vs. Post3	0.01644	0.03473	-0.01829	0.009911	27	27	1.845	26	-0.04826 to 0.01168	0.6876
Base vs. Post4	0.01644	0.04115	-0.02471	0.005466	27	27	4.521	26	-0.04124 to -0.008186	0.0011
Base vs. Post5	0.01644	0.02488	-0.00844	0.00469	27	27	1.8	26	-0.02262 to 0.005741	0.7519
Base vs. Post6	0.01644	0.03278	-0.01634	0.004258	27	27	3.837	26	-0.02922 to -0.003465	0.0064
Base vs. Post7	0.01644	0.02323	-0.006792	0.004249	27	27	1.598	26	-0.01964 to 0.006056	>0.9999
Base vs. Post8	0.01644	0.02829	-0.01185	0.004343	27	27	2.73	26	-0.02499 to 0.001278	0.101
$Pert10_R$										
Base vs. Pre	0.01969	0.02029	-0.0005999	0.004008	27	27	0.1497	26	-0.01272 to 0.01152	>0.9999
Base vs. Post1	0.01969	0.01298	0.006709	0.007452	27	27	0.9003	26	-0.01582 to 0.02924	>0.9999
Base vs. Post2	0.01969	0.05607	-0.03638	0.005344	27	27	6.808	26	-0.05254 to -0.02022	<0.0001
Base vs. Post3	0.01969	0.02668	-0.006992	0.006573	27	27	1.064	26	-0.02687 to 0.01288	>0.9999
Base vs. Post4	0.01969	0.03991	-0.02022	0.006154	27	27	3.286	26	-0.03883 to -0.001612	0.0262
Base vs. Post5	0.01969	0.0231	-0.003408	0.003743	27	27	0.9105	26	-0.01472 to 0.007909	>0.9999
Base vs. Post6	0.01969	0.03015	-0.01046	0.004406	27	27	2.375	26	-0.02379 to 0.002859	0.227
Base vs. Post7	0.01969	0.02292	-0.003227	0.004232	27	27	0.7626	26	-0.01602 to 0.009568	>0.9999
Base vs. Post8	0.01969	0.02589	-0.006201	0.002658	27	27	2.333	26	-0.01424 to 0.001835	0.2487

eTable 16: Bonferroni's multiple comparison test results comparing $Pert1_R$ to $Pert10_R$ for MoS_{ML} [m] in the older adults

Test details	Mean 1	Mean 2	Mean Diff.	SE of diff.	N1	N2	t	DF	95.00% CI of diff.	Adjusted P Value
$Pert1_R$ - $Pert10_R$										
Base	0.01644	0.01969	-0.003249	0.001499	27	27	2.167	26	-0.007847 to 0.001349	0.3955
Pre	0.02095	0.02029	0.0006582	0.003111	27	27	0.2115	26	-0.008884 to 0.01020	>0.9999
Post1	0.00586	0.01298	-0.007123	0.008802	27	27	0.8092	26	-0.03412 to 0.01987	>0.9999
Post2	0.0454	0.05607	-0.01067	0.009443	27	27	1.13	26	-0.03963 to 0.01830	>0.9999
Post3	0.03473	0.02668	0.008048	0.01128	27	27	0.7132	26	-0.02656 to 0.04266	>0.9999
Post4	0.04115	0.03991	0.001246	0.006638	27	27	0.1877	26	-0.01911 to 0.02160	>0.9999
Post5	0.02488	0.0231	0.001783	0.005093	27	27	0.3502	26	-0.01384 to 0.01740	>0.9999
Post6	0.03278	0.03015	0.002627	0.004233	27	27	0.6206	26	-0.01036 to 0.01561	>0.9999
Post7	0.02323	0.02292	0.0003155	0.003709	27	27	0.0851	26	-0.01106 to 0.01169	>0.9999
Post8	0.02829	0.02589	0.002405	0.004033	27	27	0.5962	26	-0.009965 to 0.01477	>0.9999

eTable 17: Bonferroni's multiple comparison test results comparing recovery steps to baseline in Pert2$_L$ and Pert3$_L$ for MoS$_{AP}$ [m] in the older adults

Test details	Mean 1	Mean 2	Mean Diff.	SE of diff.	N1	N2	t	DF	95.00% CI of diff.	Adjusted P Value
Pert2$_L$										
Base vs. Pre	0.04801	0.04449	0.003512	0.004828	27	27	0.7275	26	-0.01109 to 0.01811	>0.9999
Base vs. Post1	0.04801	0.06157	-0.01357	0.01348	27	27	1.006	26	-0.05434 to 0.02720	>0.9999
Base vs. Post2	0.04801	0.0001864	0.04782	0.01382	27	27	3.459	26	0.006017 to 0.08962	0.0169
Base vs. Post3	0.04801	0.01522	0.03279	0.01418	27	27	2.312	26	-0.01010 to 0.07567	0.2607
Base vs. Post4	0.04801	-0.03440	0.08240	0.01072	27	27	7.689	26	0.05000 to 0.1148	<0.0001
Base vs. Post5	0.04801	0.003906	0.04410	0.007965	27	27	5.536	26	0.02001 to 0.06819	<0.0001
Base vs. Post6	0.04801	-0.004123	0.05213	0.008075	27	27	6.455	26	0.02771 to 0.07655	<0.0001
Base vs. Post7	0.04801	0.01971	0.02830	0.006021	27	27	4.700	26	0.01009 to 0.04650	0.0007
Base vs. Post8	0.04801	0.03074	0.01726	0.004716	27	27	3.660	26	0.003002 to 0.03152	0.0101
Pert3$_L$										
Base vs. Pre	0.01969	0.02029	-0.0005999	0.004008	27	27	0.1497	26	-0.01272 to 0.01152	>0.9999
Base vs. Post1	0.01969	0.01298	0.006709	0.007452	27	27	0.9003	26	-0.01582 to 0.02924	>0.9999
Base vs. Post2	0.01969	0.05607	-0.03638	0.005344	27	27	6.808	26	-0.05254 to -0.02022	<0.0001
Base vs. Post3	0.01969	0.02668	-0.006992	0.006573	27	27	1.064	26	-0.02687 to 0.01288	>0.9999
Base vs. Post4	0.01969	0.03991	-0.02022	0.006154	27	27	3.286	26	-0.03883 to -0.001612	0.0262
Base vs. Post5	0.01969	0.0231	-0.003408	0.003743	27	27	0.9105	26	-0.01472 to 0.007909	>0.9999
Base vs. Post6	0.01969	0.03015	-0.01046	0.004406	27	27	2.375	26	-0.02379 to 0.002859	0.227
Base vs. Post7	0.01969	0.02292	-0.003227	0.004232	27	27	0.7626	26	-0.01602 to 0.009568	>0.9999
Base vs. Post8	0.01969	0.02589	-0.006201	0.002658	27	27	2.333	26	-0.01424 to 0.001835	0.2487

eTable 18: Bonferroni's multiple comparison test results comparing Pert2$_L$ to Pert3$_L$ for MoS$_{AP}$ [m] in the older adults

Test details	Mean 1	Mean 2	Mean Diff.	SE of diff.	N1	N2	t	DF	95.00% CI of diff.	Adjusted P Value
Pert2$_L$ - Pert3$_L$										
Base	0.04801	0.04985	-0.001843	0.003117	27	27	0.5914	26	-0.01140 to 0.007715	>0.9999
Pre	0.04449	0.05390	-0.009410	0.005586	27	27	1.684	26	-0.02654 to 0.007723	>0.9999
Post1	0.06157	0.02014	0.04143	0.01785	27	27	2.321	26	-0.01331 to 0.09618	0.2838
Post2	0.0001864	0.01402	-0.01384	0.01407	27	27	0.9835	26	-0.05699 to 0.02931	>0.9999
Post3	0.01522	-0.002243	0.01746	0.01406	27	27	1.242	26	-0.02566 to 0.06059	>0.9999
Post4	-0.03440	-0.01725	-0.01715	0.01234	27	27	1.389	26	-0.05500 to 0.02071	>0.9999
Post5	0.003906	0.007616	-0.003710	0.009622	27	27	0.3856	26	-0.03322 to 0.02580	>0.9999
Post6	-0.004123	0.02200	-0.02612	0.009362	27	27	2.791	26	-0.05484 to 0.002587	0.0972
Post7	0.01971	0.03925	-0.01954	0.007517	27	27	2.600	26	-0.04259 to 0.003511	0.1517
Post8	0.03074	0.04562	-0.01487	0.004319	27	27	3.443	26	-0.02812 to -0.001626	0.0196

eDiscussion

Regarding our analyses of MoS_{ML}, the results did not reveal any substantial differences with age. Both age groups showed a significant increase in MoS_{ML} in some of the first few steps post-perturbation, but there was little change in magnitude of these values with repetition. This suggests that anteroposterior gait perturbations of this nature require some mediolateral balance control but can be accommodated similarly by young and older healthy adults. It is not possible to say, based on the current work, if different magnitudes or types of anteroposterior perturbations would show a similar lack of effect with age. Sideways falls are prevalent among older adults in long-term and many falls initially directed anteriorly may lead to a lateral ground contact (Yang et al. 2016). Previous findings indicate that older age and falls history are related to lateral instability following anteroposterior perturbations to stance (McIlroy and Maki 1996; Rogers et al. 2001). However, as the current study deals with perturbations during gait, stance perturbations are obviously not directly comparable to those used in the current paradigm. A limitation that should be noted is that while our reduced kinematic model has been previously validated for the MoS_{AP} and its components (Süptitz et al. 2013), it was not validated for the MoS_{ML}. Various levels of agreement have been reported in previous studies of CoM position estimates between simplified marker sets and the full-body marker sets (Havens et al. 2018; Huntley et al. 2017; Vanrenterghem et al. 2010; Yang and Pai 2014) and these differences can stem from differences in the marker model (e.g. with and without trunk), the analysed locomotion task (e.g. level walking, cutting manoeuvres), the participant groups (e.g. healthy young adults, stroke patients) and the gait velocity and experimental setup (e.g. overground and treadmill walking). Therefore, we would caution against drawing firm conclusions regarding our MoS_{ML} results.

eReferences

Bruijn SM, Meijer OG, Beek PJ, van Dieen JH (2013) Assessing the stability of human locomotion: a review of current measures J R Soc Interface 10:20120999 doi:10.1098/rsif.2012.0999

Havens KL, Mukherjee T, Finley JM (2018) Analysis of biases in dynamic margins of stability introduced by the use of simplified center of mass estimates during walking and turning Gait Posture 59:162-167 doi:10.1016/j.gaitpost.2017.10.002

Hof AL, Gazendam MG, Sinke WE (2005) The condition for dynamic stability J Biomech 38:1-8 doi:10.1016/j.jbiomech.2004.03.025

Huntley AH, Schinkel-Ivy A, Aqui A, Mansfield A (2017) Validation of simplified centre of mass models during gait in individuals with chronic stroke Clin Biomech 48:97-102 doi:10.1016/j.clinbiomech.2017.07.015

McIlroy WE, Maki BE (1996) Age-related changes in compensatory stepping in response to unpredictable perturbations J Gerontol A Biol Sci Med Sci 51:M289-296 doi:10.1093/gerona/51a.6.m289

Rogers MW, Hedman LD, Johnson ME, Cain TD, Hanke TA (2001) Lateral stability during forward-induced stepping for dynamic balance recovery in young and older adults J Gerontol A Biol Sci Med Sci 56:M589-594 doi:10.1093/gerona/56.9.m589

Süptitz F, Moreno Catala M, Brüggemann GP, Karamanidis K (2013) Dynamic stability control during perturbed walking can be assessed by a reduced kinematic model across the adult female lifespan Hum Mov Sci 32:1404-1414 doi:10.1016/j.humov.2013.07.008

Vanrenterghem J, Gormley D, Robinson M, Lees A (2010) Solutions for representing the whole-body centre of mass in side cutting manoeuvres based on data that is typically available for lower limb kinematics Gait Posture 31:517-521 doi:10.1016/j.gaitpost.2010.02.014

Yang F, Pai YC (2014) Can sacral marker approximate center of mass during gait and slip-fall recovery among community-dwelling older adults? J Biomech 47:3807-3812 doi:10.1016/j.jbiomech.2014.10.027

Yang Y, Mackey DC, Liu-Ambrose T, Feldman F, Robinovitch SN (2016) Risk factors for hip impact during real-life falls captured on video in long-term care Osteoporos Int 27:537-547 doi:10.1007/s00198-015-3268-x
